# Cognitive Reserve and the Prevention of Dementia: the Role of Physical and Cognitive Activities

**DOI:** 10.1007/s11920-016-0721-2

**Published:** 2016-08-01

**Authors:** Sheung-Tak Cheng

**Affiliations:** 1 Department of Health and Physical Education, The Education University of Hong Kong, 10 Lo Ping Road, Tai Po, N.T., Hong Kong; 2 Department of Clinical Psychology, Norwich Medical School, University of East Anglia, Norfolk, NR4 7TJ UK

**Keywords:** Dementia, Cognitive reserve, Physical activity, Cognitive activity

## Abstract

**Purpose of Review:**

The article discusses the two most significant modifiable risk factors for dementia, namely, physical inactivity and lack of stimulating cognitive activity, and their effects on developing cognitive reserve.

**Recent Findings:**

Both of these leisure-time activities were associated with significant reductions in the risk of dementia in longitudinal studies. In addition, physical activity, particularly aerobic exercise, is associated with less age-related gray and white matter loss and with less neurotoxic factors. On the other hand, cognitive training studies suggest that training for executive functions (e.g., working memory) improves prefrontal network efficiency, which provides support to brain functioning in the face of cognitive decline.

**Summary:**

While physical activity preserves neuronal structural integrity and brain volume (hardware), cognitive activity strengthens the functioning and plasticity of neural circuits (software), thus supporting cognitive reserve in different ways. Future research should examine whether lifestyle interventions incorporating these two domains can reduce incident dementia.

## Introduction

As the human race celebrates rising longevity, societies have to confront changing disease profiles. One condition that will become more and more common is dementia (i.e., major neurocognitive disorder). Dementia is not a disease in itself; it is an umbrella term for a myriad of diseases causing cognitive impairment, of which Alzheimer’s disease (AD) is the most common form. The prevalence rate of dementia doubles approximately every 6 years from the age of 65 years, reaching 7 % in those aged 75–79 years, 12 % in those aged 80–84 years, 20 % in those aged 85–89 years, and 40 % in those aged 90 years or over [1]. Because of population aging, there is increasing interest in studying the aging brain.

Dementia is the consequence of three processes. The first of these processes is age-related, or “normal,” cognitive decline. Studies have found that most cognitive functions decline since early adulthood, and the areas affected most are those that rely on mental speed, volume of processing, and coordination efficiency, such as attention, working memory, verbal recall, reasoning, multitasking, task switching, and response inhibition [2, 3]. Knowledge of the world, people, events, and vocabulary, on the contrary, tends to be well preserved [4].

Parallel to these performance declines is a gradual loss of brain mass throughout adulthood. Certain cortical and subcortial regions involved in cognition are particularly susceptible to gray matter loss over time, including the hippocampus, caudate nucleus, putamen, and prefrontal cortex [5, 6]. Furthermore, white matter changes and injuries become prevalent after late midlife, with white matter volume declining most noticeably in the prefrontal region [5, 7]. The compromised integrity of the white matter tracts affects the connectivity between brain regions, leading to less efficient networks.

The second one is a process of pathological changes brought on by neurological diseases, leading to accelerated cognitive decline. Take AD as an example. Advances in in vivo imaging and cerebrospinal fluid (CSF) testing have opened the window for identifying the presence of beta-amyloid fibrils and plaques two decades or more before symptom onset [8]. Although whether it is appropriate or ethical to diagnose AD in the preclinical phase without effective treatment for it is a matter of debate [9], the newer research findings suggest that the brains of people with AD may be coping with the disease’s assault many years before daily functioning starts to suffer. In the preclinical phase, the person appears normal because the brain recruits additional resources to cope with injuries to networks. This leads us to the next factor and the focus of this article—the brain’s ability to redirect its resources.

## Cognitive Reserve

Cognitive reserve is a hypothetical construct that moderates the effects of age-related decline and pathological damage [10]. It refers to structural and dynamic capacities of the brain that buffer against atrophies and lesions. Tissue or functional loss at a particular brain region may be compensated by other neurons working harder in order to maintain the same level of functioning as much as possible. This compensation may happen at the “local” level, in the sense that the neighboring neurons make up for the lost neural activity from regional damage. Called “brain reserve” by some scholars [10], this type of volumetric reserve is also referred to as a passive or static model of cognitive reserve.

However, when the number of healthy or functional neurons fall below a certain threshold vis-à-vis task demands, the brain may recruit other regions and networks to help in order to maintain performance. This functional re-routing of neural circuits represents an active or dynamic model of reserve. Studies utilizing functional magnetic resonance imaging (fMRI) techniques have shown predominantly increased prefrontal, task-based activation in healthy older adults as well as those with mild cognitive impairment (MCI; i.e., minor neurocognitive disorder) and early dementia [11–13]. These over activations in the prefrontal cortex (and, sometimes, other cortices as well) are often bilateral to increase the output. They play compensatory functions because they are associated with the following: (a) under activations in posterior regions such as medial temporal lobe, precuneus, and visual cortex; (b) the inability to suppress the default mode network; and (c) improved performance across a range of tasks, including working memory, episodic memory, inhibitory control, semantic processing, processing speed, and visual perception [14]. In fact, younger adults also display similar activation patterns when cognitively overloaded. The default mode network, consisting of midline structures from the posterior cingulate/precuneus to the anterior cingulate/medial prefrontal cortex, as well as certain lateral parietal areas, is activated while the brain is not focused on external stimuli and tasks (e.g., during mind wandering or thinking about past and future) but is deactivated when it is [15, 16]. With age, the brain is less capable of suppressing the default mode network during task situations, thus interfering with task performance with irrelevant stimuli.

All of the age-related brain deteriorations mentioned above, including gray matter and white matter loss, and less efficient/functional networks are more pronounced in people who are on the way to developing cognitive impairment [17–19]. However, there is substantial heterogeneity in the clinical manifestation of dementia in individuals with similar degrees of brain pathology, which is presumably related to inter-individual differences in cognitive reserve. Cognitive reserve cannot be observed or directly measured, and proxy measures such as education, premorbid intelligence (IQ), linguistic ability, and occupational complexity are often used. Indeed, studies have found that cognitive reserve moderates the relationship between neuropathology and clinical status.

A study of 323 cognitively healthy adults found that lifetime occupational complexity (e.g., whether synthesizing and analyzing data, supervising and mentoring colleagues, and setting up and operating precise instruments), averaged up to three jobs and weighted by years on the job, was associated with *more* hippocampal and whole brain atrophy, after controlling for global cognition, apolipoprotein E ε4 (APOE4) status, and cardiovascular risk factors [20]. Another analysis from the same sample showed that educational level attenuated the age trend in CSF biomarkers of AD such that those with higher (especially university) education had lower tau concentrations and tau/Aβ_42_ ratios in the CSF [21]. Another study following 245 adults for an average of 11 years operationalized cognitive reserve in terms of reading level, vocabulary, and years of education combined. Interestingly, the researchers found that the left entorhinal cortex volume at baseline predicted time to MCI symptom onset in those with low cognitive reserve only, controlling for baseline cognitive reserve and APOE status. Among those with high cognitive reserve, there was no connection between the degree of entorhinal atrophy and time to symptom onset [22•]. Similarly, in a large study involving 2400 individuals, an additional year of education was associated with a 13–18 % reduction in the likelihood of receiving a diagnosis of AD at the clinical examination most proximal to, and within 1 year of, death, after controlling for neuropathology examined at autopsy (e.g., the extent of plagues and/or tangles) and history of stroke [23]. This condition is sometimes referred to as *Alzheimer’s disease without dementia*, as the individual has the pathological condition indicative of AD but is either asymptomatic or has borderline symptoms that do not cross the threshold for clinical diagnosis.

Cognitive reserve is hypothesized to empower the brain to tolerate atrophies and insults and, as a result, delay symptom onset. At the same time, because the brain is more compromised by the time that symptoms appear, those with higher cognitive reserve tend to experience a more rapid course of decline afterward. At this point, there is no evidence that cognitive reserve would postpone mortality [24, 25], and hence, a pattern consistent with compression of morbidity is expected [26]. To what extent a high cognitive reserve would delay symptom onset is not at all clear, but any noticeable delay, be it 1 or 2 years, would translate into tremendous public health benefits by reducing the prevalence of dementia.

While characteristics such as education and intelligence are relatively stable since young adulthood, there is increasing interest in the role of leisure activities in building up cognitive reserve, which will be the focus of this article. The emphasis will be on physical and cognitive activities as these have received the strongest support in the literature in terms of the prevention of dementia. The hypothesis proposed is that physical (especially aerobic) exercise and cognitive activity work together to protect brain health in general, thus reducing the loss of brain mass with age and strengthening compensatory circuits primarily through enhancing executive function.

## Physical Activity

A recent analysis examined the relative contributions of seven potentially modifiable risk factors (i.e., diabetes mellitus, midlife hypertension, midlife obesity, depression, physical inactivity, smoking, and low education) to AD disease burden. Diet was excluded because of the difficulty in aggregating data from diverse dietary factors. Physical inactivity was defined as not doing 20 min of vigorous activity for at least 3 days per week or 30 min of moderate activity for at least 5 days per week. Low educational attainment was defined as having lower secondary education or less. By calculating the population attributable risks, the authors estimated that, for the world as a whole, low education and physical inactivity were the two most important modifiable risk factors, accounting for 19 and 13 % of AD prevalence, respectively, or nearly 11 million cases combined in 2010. The contribution of low education was not as pronounced in the developed regions such as the USA and Europe, where physical inactivity accounted for over 20 % of the prevalence [27••].

Longitudinal studies controlling for risk factors such as lipids, hypertension, diabetes, obesity, history of stroke, depression, smoking, educational level, APOE status, and so on generally confirm the importance of physical activity in reducing dementia risk [28]. For instance, in a study of 2492 older Germans, engagement in any additional physical activity on a regular basis was associated with a 20 % risk reduction in terms of dementia onset over the next 4.5 years. No effect was found for cognitive activities [29]. Such medium follow-up interval is typical in this literature, but a longer interval would be needed to examine the long-term effects of physical activity as a potential preventive agent. A study of 803 older adults residing in a suburban area of Japan found that those with self-reported leisure-time physical activity of at least 1 day per week had a 41 % reduction in the risk of developing AD (but not other types of dementia) over the next 12 years, compared with those who were less active. Other types of physical activity, such as household chores, were not examined in this study [30]. That any physical activity, when compared with a sedentary lifestyle, could reduce the odds of dementia or dementia-related mortality also received support from other longitudinal studies of older adults [31–33]. The magnitude of risk reduction reported in the Japanese study was consistent with the effect size found in a recent meta-analysis of nine other studies [34].

It is not difficult to understand why light physical activity would be beneficial for older adults. Yet, even physical activity of suboptimal amount and intensity at midlife appears to be similarly beneficial. A study following over 1200 Swedish adults for 21 years found that engaging in leisure-time physical activities of moderate intensity for at least twice a week at midlife was associated with a 53 and 65 % reduced chance of developing all-cause dementia and AD, respectively, in old age, compared with less frequent activities [35]. While studies tend to use the amount/intensity of physical activity at one point in time as the predictor of future dementia risk, the implicit assumption is that this one-time measure reflects a more stable habit. Indeed, studies have also found that an increase or decrease of physical activity over time changes the odds of getting dementia in the expected direction [36•, 37].

Longitudinal studies, however, do not prove causality. Those with an underlying disease process, though not yet diagnosable with dementia, may start reducing activity long before symptom onset. Randomized controlled trials (RCTs) are needed to demonstrate causality. However, the follow-up intervals in RCTs are usually not long enough to make any meaningful assessment of potential effects on dementia incidence. One study has attempted to address this question. Three hundred eighty-nine Hong Kong older adults with MCI were randomized to a Tai Chi group and a toning and stretching exercise group (control). Tai Chi, a mind-body exercise, also has an aerobic component and improves cardiorespiratory fitness [60]. After some initial training, participants were instructed to continue practicing the exercise at least three times a week, each for a minimum of 30 min, for a total of 12 months from the start of intervention. Experimental effect in terms of conversion to dementia was found for the 54 and 78 % of the Tai Chi and control participants, respectively, who adhered to the regime throughout, but not for the whole sample (note the higher attrition in the former). Among these completers, 4 and 17 % of the Tai Chi and the control group, respectively, progressed to dementia by the end of the year, representing an 80 % risk reduction in multivariate models [38]. However, because of differential attrition rates between the two groups and because of a lack of treatment effect in the intent-to-treat analysis, the results have to be taken with caution.

Why should physical activity work in reducing the risk of dementia? It is likely that physical activity protects brain health via a number of pathways. Although the benefits of exercise have primarily been demonstrated in animal studies previously, more recent studies, including RCTs, have been able to replicate these findings in humans [39, 40]. In a nutshell, physical activity protects brain health and fuels neuroplasticity by reducing the likelihood of vascular diseases and improving cerebral perfusion (e.g., plaque deposits in arteries, atherosclerosis, hypertension, and stroke) [41–47], improves respiratory function [48], stimulates growth factors particularly brain-derived neurotrophic factor (BDNF) and insulin-like growth factor-1 [49–52], and downregulates oxidative stress and inflammatory responses [43, 44, 53]. It also reduces the brain’s exposure to neurotoxic factors, including beta-amyloid and excessive glucose [41, 54–56]. For these reasons, it is no wonder that the cognitive benefits of physical activity appear to be limited to aerobic exercise [57–59]. At the same time, it is important to note that many physical activities also have mental stimulation properties such as those that require eye-hand coordination and visuospatial memory, thus further augmenting their effects on cognitive functioning.

The association between physical activity and amyloid burden in the brain deserves special attention because of the latter’s significance for AD pathology. A few studies have demonstrated, on a cross-sectional basis, a negative association between physical activity level and beta-amyloid load in the brain, whether measured by Pittsburgh Compound B (PIB) positron emission tomography (PET) or CSF analysis, in older adults, although this association was not consistently found [61]. Liang and colleagues reported that cognitively intact older adults who performed 30 min of moderately intense exercise for at least 5 days a week had lower PIB uptake or higher levels of CSF Aβ_42_ (i.e., more amyloid clearance from the brain), compared with less active individuals; however, no relationship between physical activity and CSF tau was found after controlling for cardiovascular risk factors, APOE4 status, and depressive symptoms [54]. Brown and colleagues found that a relationship between high levels of physical activity and reduced PIB uptake existed even among physically active, but APOE4-positive, individuals [55]. However, these cross-sectional relationships are difficult to interpret because a lack of, or decrease in, activity may be the result of brain changes. Without longitudinal studies, it is difficult to judge whether physical activity actually downregulates beta-amyloid production, especially in at-risk individuals.

Despite certain ambiguities in the state of the field, the broader picture remains supportive of the neuroprotective effects of physical activity. Importantly, the improvements in cognition attributable to physical exercise appear to be mediated by gains in brain volume and network connectivity [46], which in turn are partially mediated by increases in perfusion and growth factors [46, 47, 52, 62]. Two recent systematic reviews support the positive effects of long-term physical activity on white matter volume and white matter integrity [63] as well as gray matter volume [64], although the effects on the former were small and inconsistent. For instance, one RCT reported no improvements in white matter integrity in older adults after a 1-year walking exercise intervention, compared with those having stretching exercise; both groups had three 40-min sessions per week led by an exercise trainer. However, gains in cardiorespiratory fitness in terms of maximal oxygen consumption (VO_2_ max) in the walking group were moderately correlated with white matter integrity (as measured by fractional anisotropy from diffusion tensor imaging) in the frontal and temporal lobes.

The benefit of physical activity on slowing age-related decline in gray matter is not limited to cognitively healthy adults but exists for those with MCI or dementia as well [65, 66]. Thus, it is never too late to increase physical activity. The hippocampal volume and its relationship to long-term physical activity have been a subject of much research interest partly because of neurogenesis in the dentate gyrus section throughout adulthood [67] and partly because hippocampal atrophy is one of the hallmarks of early AD. An RCT comparing moderate-intensity aerobic exercise with stretching and toning exercise of 1-year duration, 3 days a week, reported a 2 % increase in bilateral hippocampal volume in the former, versus a 1.4 % decrease in the latter, representing a 1–2-year reverse in age-related decline in hippocampal volume. Furthermore, the increase in hippocampal volume in the aerobic exercise group was associated with changes in VO_2_ max and serum BDNF [62]. Other studies have found positive effects on brain regions other than the hippocampus. In another RCT, older adults who participated in exercise interventions for 6 months showed increases in physical activity, which in turn was correlated with increased gray matter volume in the prefrontal and the cingulate cortex, compared with those without intervention [68]. Another intervention study suggested that the effects of aerobic exercise on gray and white matter volumes may be specific to older, but not younger, adults [69].

Erickson and colleagues suggest that the main neurological benefit of physical activity is the preservation of neurons and synapses in areas where age-related atrophy is most apparent, namely, the prefrontal cortex and the hippocampus [64]. Nevertheless, this body of literature is mostly correlational, and only a handful of studies have employed longitudinal or experimental designs. Despite such a limitation, these neurological findings corroborate the results of RCTs of cognitive outcomes. Although the overall effect sizes tend to be small, executive functions, attention, processing speed, and memory are the cognitive domains most responsive to physical activity interventions [51, 70, 71]. These domains happen to correspond well with the same brain regions where volumetric advantages associated with physical exercise have been found [64].

The effect on executive function is noteworthy as those with poor executive function, probably because of deficits in goal-directed behavior, performance monitoring, and inhibition of prepotent responses, tend to reduce physical activity over time, leading to a vicious cycle [72]. In an RCT comparing a 1-year resistance training program to a balance and toning control in older women, the long-term maintenance of physical activity, up to 1 year post-intervention, was predicted by gains in executive function during the intervention [73].

## Cognitive Activity

The London taxi driver study showing that taxi drivers (who had to navigate through the streets of London purely from memory, per license requirements) had larger posterior hippocampus than bus drivers (who drove fixed routes) [74] was seminal in suggesting the effect of cognitive activity on neurogenesis in the hippocampus. Yet, experimental studies in humans are few. An exception was a recent study comparing a virtual-reality spatial-navigation training, conducted every other day over a 4-month period, with a leisure walking exercise on treadmill as control. Results showed maintenance of hippocampal volume for up to 4 months after the training for both older and younger adults who were in the navigation training group, compared to a decline in hippocampal volume in the walking group [75].

A prospective study showed a moderate correlation between engagement in complex mental activities and both volume and reduced atrophy rate of the hippocampus after 3 years. However, only 37 older adults were included in the study [76]. On the other hand, there was no correlation between past cognitive activity, measured retrospectively, and gray and white matter volumes in a large sample of older women (*N* = 393) [77].

Other studies attempted to study the relationship of cognitive activity with AD biomarkers. In an interesting study [78], 65 cognitively intact adults provided data on current cognitive activity as well as cognitive activity at 6, 12, 18, and 40 years old on a retrospective basis. Only past cognitive activity summed across the previous ages was correlated inversely with PIB retention. Physical activity as measured by calorie consumption in a recent 2-week period was also negatively correlated with PIB retention, but the correlation disappeared after controlling for past cognitive activity. Moreover, when these cognitively healthy participants were divided into tertiles according to past cognitive activity, the least active ones had beta-amyloid levels similar to that of ten age-matched participants with AD, whereas those most active had levels similar to that of 11 young adults. Although it is tempting to read the data as supporting the protective role of lifetime cognitive activity, the data are actually difficult to interpret as the inactive individuals were *asymptomatic* at similar amyloid levels to those with AD. The data may be interpreted as *low* cognitive activity being protective as the higher amyloid burden in these individuals did not cause symptoms, unless there were longitudinal data showing that these individuals were on an impending decline into dementia. In addition, a study of 186 older adults did not find any correlation between past/current cognitive activity and a number of biomarkers, including PIB uptake, glucose metabolism (fluorodeoxyglucose PET), and hippocampal volume [79], while another study of 118 older adults found that lifetime cognitive activity was associated with less PIB uptake in APOE4 carriers [80].

At best, the available evidence is mixed and inconclusive as to whether cognitive activity has direct impacts on brain structure and physiology. One difficulty with this line of research is accounting for the variety of cognitive activities and the relationship of each activity to neuropathology. For instance, while challenging spatial navigation conducted frequently enough (e.g., what London taxi drivers do on a daily basis) may stimulate noticeable neurogenesis in the hippocampus, we are not entirely sure what playing chess would do to the brain. Moreover, activities cross domains; that is, many cognitive activities have social components (e.g., playing bridge), and there is no easy way to separate the effects of the different components (the same may be said of physical exercises with mentally stimulating properties). For example, Leung and colleagues classified the following as primarily cognitive activities: reading, using computer, board/card games, mahjong, participating in forums or discussions, writing, calligraphy and painting, handicraft, playing musical instruments, investment in stock market, and gambling [81]. Not every researcher would agree with this list; unfortunately, there is not a standard list of activities to use in research (some studies include singing, going to museum, etc.). In any case, the point is that the list includes activities of quite a diverse nature in terms of their mental stimulation properties. Researchers typically ask respondents to indicate the frequency of engaging in each activity and then sum the ratings up to form a total activity score. But, since the mental stimulation property of each activity is not the same, much noise is introduced into the measure with people who have the same score but very different degrees and types of mental stimulation in everyday life. Passive cognitive activities, such as watching television, appear not to be helpful. Studies have actually shown watching television to increase the odds of dementia and mortality [82–84], partly because those who watch television a lot tend to be sedentary.

In addition, while there are established ways to rate or define the intensity of physical activity, no such methods exist for cognitive activity. What constitutes moderate- or high-intensity cognitive activity, for example? Thus, when there is a negative finding between cognitive activity and brain measures, it is not sure whether the wrong measure of cognitive activity was used or whether the nil finding should have been expected.

Despite the fact that there exists no strong evidence of cognitive activity on brain health, cognitive activity is often found, in large-scale longitudinal surveys, to be a stronger, even the sole, predictor of cognitive decline and incident dementia when compared with physical and social activity [81, 85–88], although there are exceptions [89–91]. As individuals who are physically active also tend to be intellectually active [92], the effect of physical activity may be difficult to parse out from the effect of cognitive activity. Typically, a high level of cognitive activity is associated with a roughly 50 % reduction in the risk of developing dementia in the next 4–5 years, after controlling for APOE4 status, cardiovascular risk factors, educational level, and so on. These findings are consistent with studies suggesting that for those who subsequently develop dementia, a high level of premorbid cognitive activity is associated with delayed symptom onset, followed by an accelerated course of decline [93–95], as is predicted by the cognitive reserve hypothesis. Such a relationship has not been observed for physical activity [94].

Despite these research findings, it would be premature to conclude that cognitive activity is necessarily more important than physical activity. A recent study followed 864 older adults over 6 years. Participants were divided into four groups based on a median split on the frequency of habitual cognitive activity and whether the participant obtained moderate-intensity exercise from everyday activities. Those with low cognitive activity and without moderately intense physical activity were the reference group. The results showed that those without physical activity, despite being high in cognitive activity, did not differ from the reference group in terms of the odds of incident MCI during the follow-up period. But, among those with any physical activity, the odds were lower, being 48 % lower in those with low cognitive activity and 80 % lower in those with high cognitive activity [91]. The data suggest that physical activity is essential, while cognitive activity adds further benefits. More research is needed to replicate this finding.

Again, RCTs are needed to ascertain causality. However, RCTs of cognitive leisure activity are difficult to do. It is difficult to pick one leisure activity (or several ones forming a package) among such a wide variety that would appeal to, and be maintained by, study participants who had been “inactive cognitively.” And, it would be unrealistic to do an RCT for every single activity (e.g., chess, crossword puzzle). While inactivity at baseline is an ideal precondition for testing the effect of adding activity to participants’ lives, exactly what constitutes cognitive inactivity is not clear. To date, RCTs on cognitive leisure activity have been limited to samples with cognitive impairment only [96, 97]. And, there is some evidence that mahjong can modulate the trajectory of decline in people with mild-to-moderate dementia in the medium term, as evidenced in the maintenance of Mini-Mental State Examination and a slower rate of increase in the Clinical Dementia Rating Sum-of-Box score up to 9 months from baseline [98•, 99].

It should be noted that the protective effect of cognitive activity may be disguised under other names in the literature. For instance, low educational level accounts for more AD cases worldwide than physical inactivity [27]. While advanced educational attainment is considered a proxy of cognitive reserve, it probably derives its benefits from enabling a lifetime of higher-level cognitive activity [25, 93], partly via holding cognitively stimulating jobs [100]. An occupation with complex duties may be characterized as an enriched environment for cognitive stimulation on a day-to-day basis.

Another related topic concerns whether speaking more than one language protects one against dementia, and the research evidence to date is mixed [101–103]. Gold’s review [104] suggests that the benefits of lifelong bilingualism appear not to be related to the possession of another language per se. The benefits are more likely due to the neural effects of switching between languages and inhibiting one while using the other. Frequent engagement in this activity leads to reduced neural costs (i.e., requiring less efforts, as evidenced by lower activation in the prefrontal executive control circuitry) in mental switching that are generalized to non-language domains. In this sense, speaking more than one language in daily life (but not simply acquiring another language) is an intellectual activity that strengthens executive control.

The review so far has focused on leisure activities that are cognitively stimulating. As leisure, these cognitive activities have the properties of being interesting, pleasant, and motivating to the individual and are therefore more likely to be sustained over time [97, 105]. Group activities facilitate social integration, which may also be cognitively protective [90, 106]. Moreover, picking up abandoned hobbies again may bring back memories of old times when the activities were enjoyed together with family and friends, making these activities more stimulating (the same may be said of other kinds of activities).

While the exact neurocognitive effects of many leisure activities are not known, there are plenty of cognitive training programs tailor-made to stimulate specific functions or abilities, such as memory, attention, speed of processing, and executive function. This is a large body of literature that will not be reviewed here in detail, and readers are referred to other expert reviews [107, 108], including Gates’ article earlier in this journal [109]. Typically, cognitively healthy older adults, after training, can behave like younger adults at baseline (without training), although the training effects are noticeably weaker in those with cognitive impairment. Owing to the growing interest in this area, some researchers have attempted to evaluate the relative effects of cognitive training and physical exercise, and the results tend to suggest that adding physical exercise to cognitive training does not yield additional cognitive benefits [96, 110, 111], suggesting a possible ceiling effect in these interventions.

Also echoing the above observation are preliminary results from the Finnish Geriatric Intervention Study to Prevent Cognitive Impairment and Disability (FINGER), a large-scale RCT combining dietary regime, physical exercise, and cognitive training in a sample of about 1200 adults aged 60–77 years at baseline. Those in the control group received regular health advice. Both intervention and control participants showed improvements in global cognition as measured by a comprehensive neuropsychological battery, with a larger improvement in the former equivalent to a 0.02 SD differential in the first year and a 0.04 SD differential in the second year. When subcomponents of the battery were analyzed separately, significant treatment effects were found for executive function and processing speed, but not for memory [112••]. Overall, the effect size was very small, suggesting that there may be a ceiling effect for lifestyle interventions and that the effects of components do not necessarily add up.

A long-standing issue with cognitive training is the lack of transfer to untrained tasks (i.e., tasks that were not directly trained), though tasks that are more similar to the trained ones have more success. The lack of “far and broad transfer” (e.g., from memory training to gains in verbal reasoning) means that the gains in training would not lead to meaningful improvements in everyday functioning, and so, they were of limited relevance to people’s lives. Moreover, many cognitive training programs, by virtue of the stimuli used or the repetition of the same stimuli over time, fail to capture people’s attention for long periods or to maintain their interest and motivation in the long run. Thus, these programs tend to lack ecological value. In addition, for older adults with relatively limited cognitive resources, let alone those with cognitive impairment, learning a novel intellectual exercise may prove to be difficult [113].

An emerging view is that many of these training programs may have targeted the wrong domains. In particular, the early focus on memory and memory strategy training (e.g., using visualization techniques as memory aids) has produced disappointing results in relation to transfer [107, 108]. More recently, there has been growing interest in the efficacy of working memory training (i.e., exercises that require holding and manipulating information mentally for brief periods). The rationale is that with age, many performance issues, including reasoning, mental switching, multitasking, and decision making, have deficits in working memory in common. While researchers have attempted to summarize the effects using meta-analyses, the results appear to be very sensitive to which studies were included and the way that the data were coded, leading to conclusions that working memory training has small far-transfer effects equivalent to a 0.20 SD between intervention and control participants [114, 115], as well as nil effects [116]. Another meta-analysis of computerized training programs found no effect of working memory training on untrained domains [117].

The meta-analysis by Karbach and Verhaegen [114] also included a separate analysis of studies training executive control processes, including speed of processing, task switching, updating, and inhibitory control, based on studies listed in PsycINFO and PsycARTICLES only (a potential flaw). They found slightly smaller far-transfer effects pooled across these training programs than that found for working memory training, but the difference in effect size did not reach statistical significance.

More research is needed to ascertain the far-transfer effects of executive function training. Equally important, however, are studies that reveal why executive function training should work, if it does, by examining how the brain changes with it. Studies in this area are only beginning to emerge. In healthy older adults, but not those with MCI [118], working memory training is associated with a decreased prefrontal activation when performing *n*-back tasks that require participants to continuously update information while comparing the new stimuli presented to the one *n* step (i.e., number of steps) backward, with a higher *n* demanding a higher cognitive load of processing. Whereas Heinzel and colleagues [119] reported such a finding in a low load condition (1-back), Vermeij and colleagues [118] found it in a medium load condition (2-back). The decreased activation, relative to performance, was taken to imply improved network efficiency. However, none of these studies had a control group and none assessed untrained tasks. Another study included a matched control, and the results suggested transfer of reduced neural activation to an untrained updating task as well as transfer of performance to other neuropsychological measures of executive functioning and fluid intelligence [120]. RCTs with larger samples are needed in future research.

Probably, very few persons, if any, would do *n*-back training on a regular basis in order to stem cognitive decline. Moreover, the laboratory tasks used to assess transfer often have little relevance to everyday functioning. Nevertheless, these preliminary studies suggest that certain training activities targeting executive function may improve the efficiency of prefrontal networks, potentially releasing resources for compensatory functions when the brain is overloaded. The fact that the improved neural efficiency is found at low-to-medium cognitive load (i.e., 1- to 2-back only, but not 3-back) suggests a possible limit to the capacity of cognitive reserve that may be just suitable for handling most everyday functional tasks. More research is needed to find out the kinds of training task or activity that will most likely strengthen compensatory resources. These investigations may have implications for guiding the kinds of leisure cognitive activity that we choose in daily life.

## Concluding Comments

In the absence of a vaccine or a disease-modifying agent against dementia, nonpharmacological interventions such as lifestyle modification remain the only feasible, population-based approach to preventing dementia. Leisure activities are generally low-cost options with physical and cognitive health benefits. Those that are culturally valued [97] will likely draw widespread participation and family/peer involvement to increase the likelihood of long-term maintenance [105]. While it would be optimal to start adopting healthy practices early in life, the available research evidence suggests that it is not too late to increase physical and cognitive activity in old age.

The current state of knowledge regarding what cognitive activity actually does to protect the brain against dementia is very nonspecific. Thus, we need to refer to the literature on cognitive training for clues. Echoing the literature in clinical neuroscience about the compensatory role of prefrontal networks, studies of cognitive training suggest that executive function training may be strategic, although the literature is not clear whether certain executive functions (such as working memory) are more crucial than the others. At the same time, the protective effect of occupational complexity may also be related to the demands for executive function in challenging jobs. Thus, it is possible that not all cognitive activities are equal in terms of offering protection against dementia. In the long run, training programs need to address barriers to real-life implementation. Video games that are fun to play with may be one of the most effective cognitive exercises [121].

The comparisons between cognitive activity and physical exercise, though of academic value, will likely have little practical value as the general population will be recommended to do both. Without ignoring the mental stimulation properties of many physical activities, the most sensible conclusion that one can draw from the current state of the literature is that *while physical activity, especially aerobic exercise, supports neuronal structural integrity and preserves brain mass (hardware or static brain reserve), cognitive activity strengthens the functioning and plasticity of neural circuits (software or dynamic cognitive reserve)*. Without healthy neuronal structures, one’s ability to participate in and respond to cognitive training is undermined. At the same time, cognitive function, especially executive processes, appears to enhance adherence to physical activity program [72, 73]. The two go together. Thus, the prospect of dementia prevention depends on population-based interventions that incorporate these multiple domains. Although the preliminary effect size of the FINGER trial was small, it should be borne in mind that a small effect in the general population may be translated into large public health benefits. The field awaits results from longer follow-ups to see whether such lifestyle interventions can truly reduce the likelihood of dementia.
